# Experimental dataset on the thermo-mechanical response of an energy piled wall section

**DOI:** 10.1016/j.dib.2025.112064

**Published:** 2025-09-12

**Authors:** Luis Villegas, Raul Fuentes, Guillermo A. Narsilio

**Affiliations:** a Department of Infrastructure Engineering, The University of Melbourne, Grattan Street, Parkville, VIC 3010, Australia; b Institute of Geomechanics and Underground Technology, RWTH Aachen University, Mies-van-der-Rohe-Straße 1, 52074, Aachen, Germany

**Keywords:** Retaining wall, Shallow geothermal, Energy structures, Energy piles

## Abstract

The investigation of geotechnical structures as heat exchangers for ground source heat pump (GSHP) systems has been extensive, mainly aiming at reducing initial capital costs of implementing renewable energy sources for space heating and cooling. Despite advancements in understanding the thermo-mechanical response of energy pile foundations, industry adoption lags behind academic developments. To bridge this gap and broaden the application of this technology to other geotechnical structures, there is a need for reliable, easy-to-use methods and confidence building through demonstration projects. This work presents a thermo-mechanical dataset from a pilot project of three energy piles within a retaining wall system at a Metro station in Melbourne, Australia. The data encompasses heat carrier fluid flow rate and temperature, station (air) temperature, soil temperatures at two depths, and temperature and uniaxial strains at different depths and locations across two piles. Unlike previous datasets focused on single piles, this dataset captures both single and multiple (three) simultaneously activated piles within a piled retaining wall, providing a better representation of an energy wall system. Beyond the raw data interpretation and model validation, the dataset can be used to evaluate the system's thermal performance and develop analytical methods needed in the industry.

Specifications Table

**Table:** 

Subject	Geotechnical engineering, Renewable energy
Specific subject area	Experimental flow rate, temperature, and strain measurements in a field-scale campaign in a piled wall section.
Data format	Raw, Analysed
Type of data	Spreadsheet - M.S. Excel file, Figures – PDF and SVG format
Data collection	The data were collected during the monitoring of five thermal tests involving either one or three reinforced concrete piles, each 40 m in length and 1.05 m in diameter. A thermal response test (TRT) GeoCube unit was used to heat and circulate the carrier fluid (plain water). The flow rate and fluid temperature were measured by a built-in flow meter and four temperature sensors (i.e. two pairs at the inlet and outlet) with ±0.2 °C accuracy, recorded at 10-minute intervals. The soil temperature was measured at two levels (8 and 16 m depth), along a 2 m length, using subhorizontal boreholes equipped with six sensors (Geokon, 3810), each with ±0.2 °C accuracy. The pile temperature and strains were measured using vibrating wire rebar strainmeters (Geokon, 4911–4). The sister bars have *a* ± 0.2 °C and ±0.25 % of full scale (F.S.) accuracy. Measurements from both soil and piles sensors were recorded every 30 min.
Data source location	Data was collected during the construction of the State Library Station for the Metro Tunnel Project, Melbourne. Data is stored in the University of Melbourne server/database (Parkville – VIC, Australia).
Data accessibility	Repository name: University of Melbourne Figshare Archive Data identification number: 10.26188/26,426,755 Direct URL to data: https://figshare.com/s/843eefdb6acbfa2fe7ee
Related research article	L. Villegas., R. Fuentes, and G.A. Narsilio, The effect of thermoactivated pile spacing on the thermo-mechanical response of energy soldier piled walls - Experimental study and initial numerical observations. Canadian Geotechnical Journal, 2025. Accepted (Accepted). [1]

## 1 Value of the Data

• The experimental setup is the first thermo-mechanical case study that comprises more than a single energy pile observation within a retaining wall. The dataset includes different thermal operation modes and multiple thermally activated piles, which is attractive for practitioners and researchers.
• The data demonstrate reduced temperature changes and induced strains under intermittent operation (tests 2 and 4) compared to continuous operation (tests 1 and 3); differences between a short and a longer activation period (tests 1 and 5), and provide opportunities for comparison with single piles, both within this study or against others.
• This dataset may support future research on energy wall design by enabling benchmarking of multi-pile thermo-mechanical responses in retaining systems with similar characteristics, and by facilitating the validation and development of analytical methods that account for variables not yet considered.

## 2 Background

The pilot energy pile wall section is the outcome of a collaborative initiative between industry and academia to promote the implementation of energy geostructures and investigate their thermal and thermo-mechanical performance. Initial numerical evaluations helped to identify the potential of using soldier pile walls as ground heat exchangers [2,3], which was later further validated through thermal response tests [4,5] and used for parametric numerical analysis [5,6]. The piles were installed as part of the retaining structure of a new metro station. The use of this technology has been broadly studied on pile foundations, and their thermo-mechanical response seems to have reached a mature level of understanding in terms of their structural short-term design [7]. Nevertheless, the structural design of the retaining piles differs from that of foundations. Their design depends on the bending moments and shear forces rather than axial loads and stresses. Furthermore, their thermo-mechanical boundaries differ from those surrounded wholly by soil. On one side is the soil, and on the other, there are the lateral supports, both constraining the pile's free thermal expansion or contraction. Although energy walls have been operating for over three decades [8], there is a limited number of reported observations on piled retaining walls [[9], [10], [11], [12], [13]]. Moreover, most of these observations are restricted to a single activated pile, limiting the available evidence for understanding the thermo-mechanical behaviour of energy piled walls. This dataset provides measurements from multiple closely spaced activated piles within a retaining wall, a more representative condition for energy wall systems [1,14].

## 3 Data Description

**Fig. 1:**
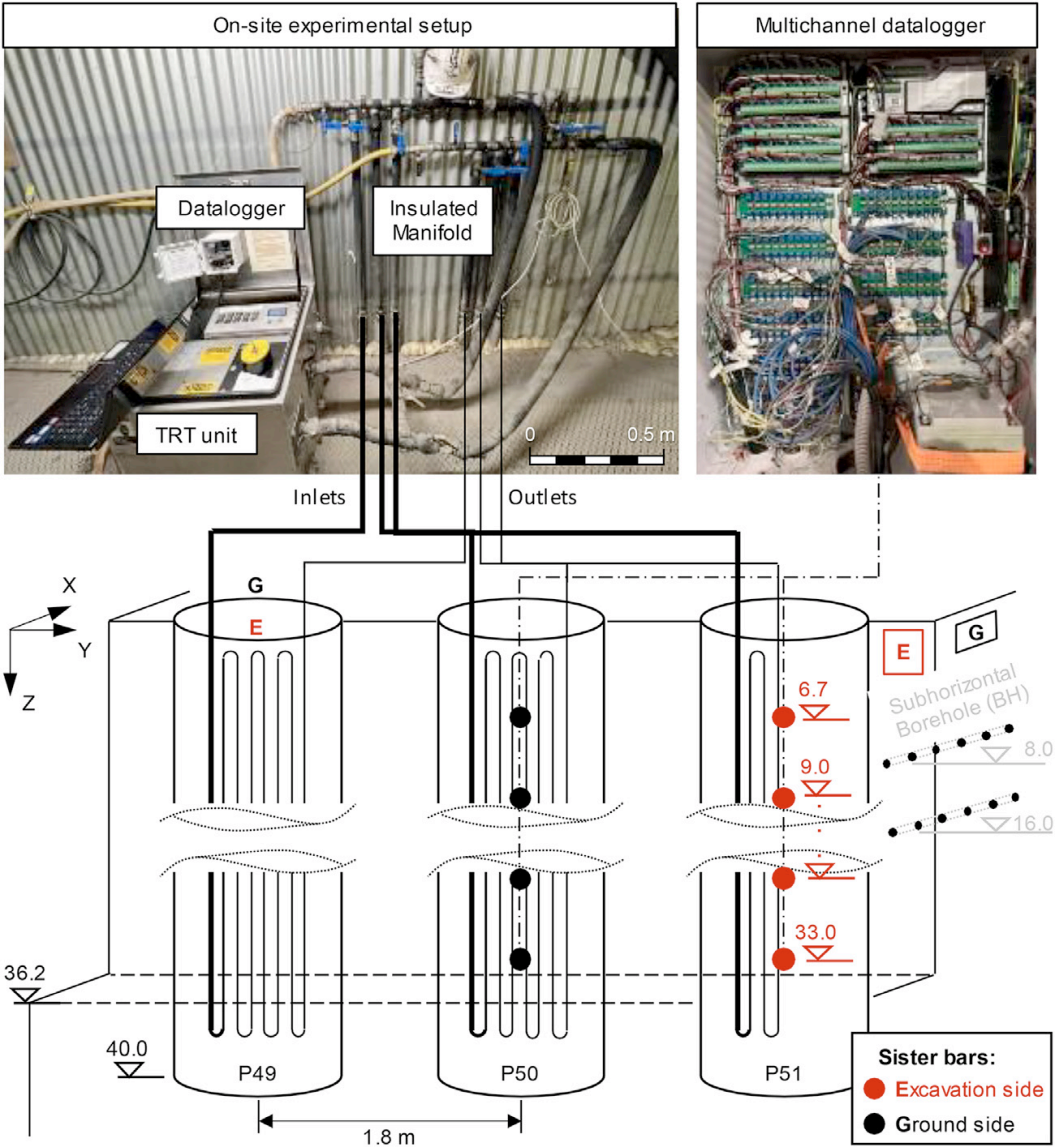
Experimental setup.

**Fig. 2:**
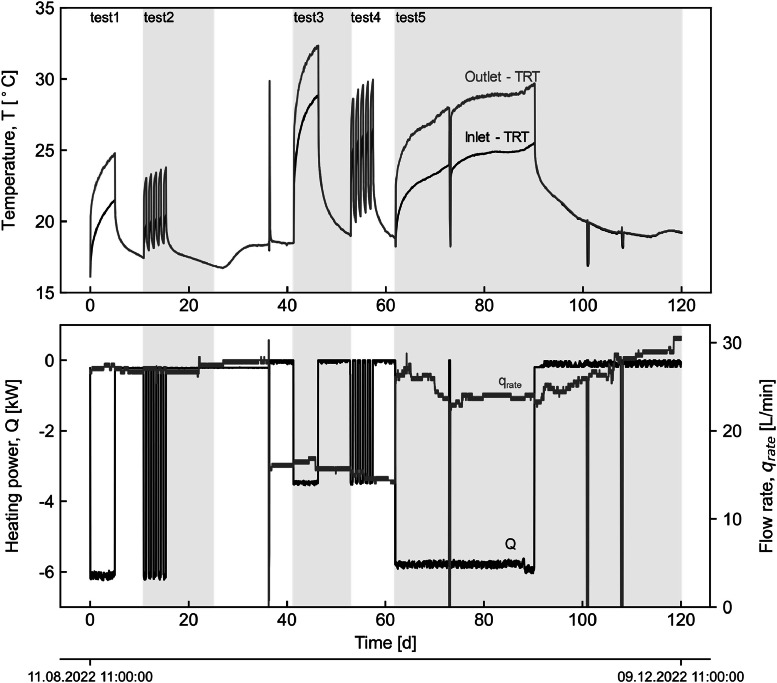
TRT unit measurements: inlet (to the TRT unit, coming from the ground loops) and outlet (from the TRT unit, entering the ground loops) fluid temperatures; heating power Q; and fluid flow rated q_rate_.

**Fig. 3:**
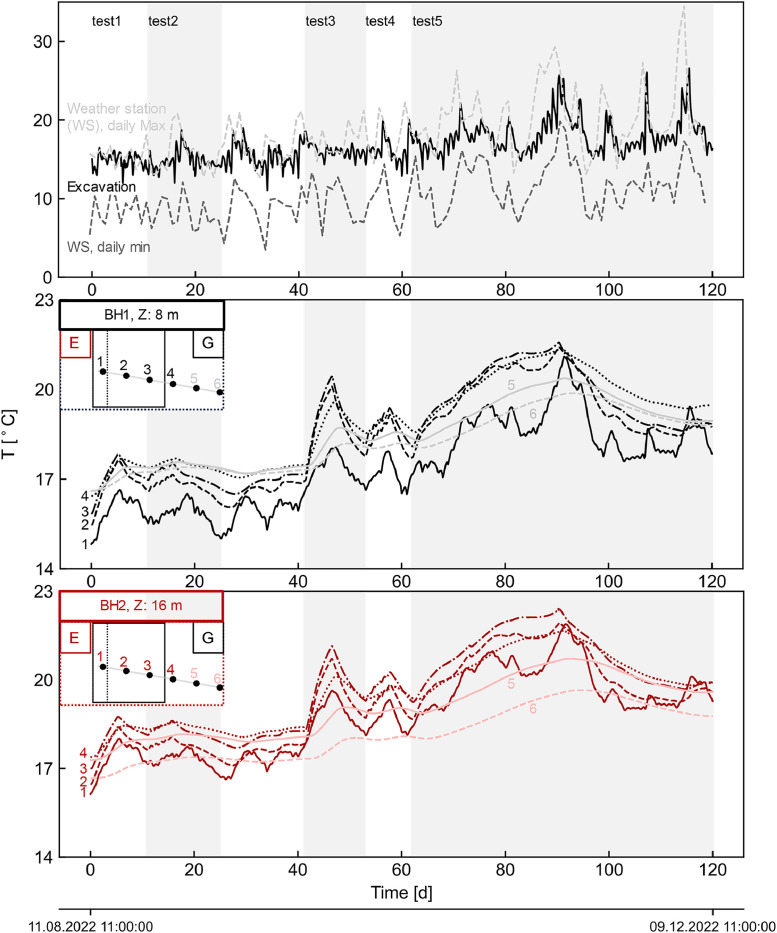
On-site excavation and outside external air temperature from weather stations (the latter data obtained from [15]), and subhorizontal boreholes soil temperature records.

**Fig. 4:**
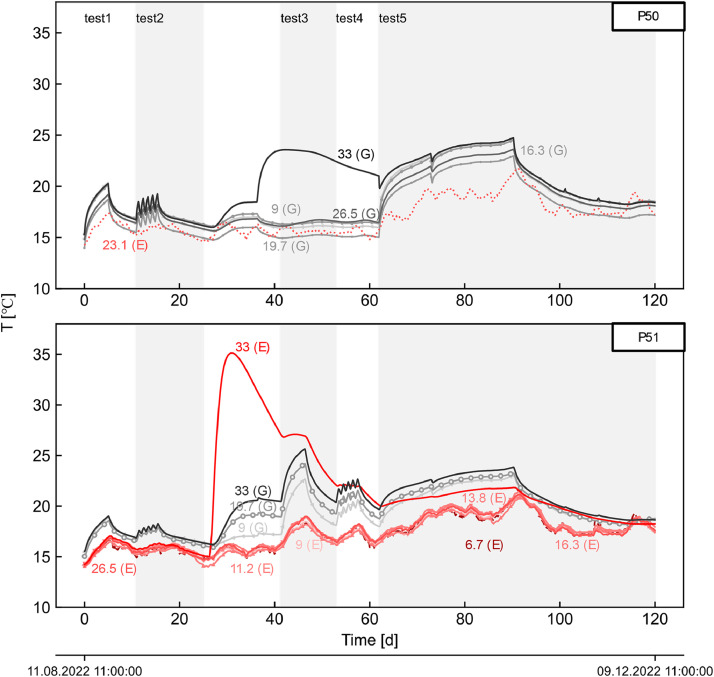
Piles No 50 (top) and 51 (bottom), temperature records on the ground (G) and excavation (E) sides at different depths (shown in meters).

**Fig. 5:**
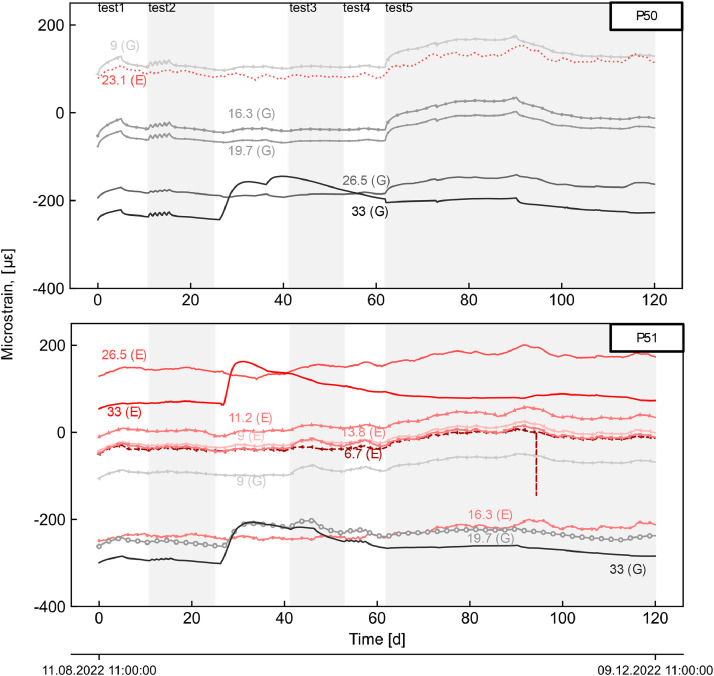
Piles, uniaxial strains records on the ground (G) and excavation (E) sides at different depths (shown in meters).

The dataset describes the recorded measurements obtained over four months of thermo-mechanical testing on a pilot energy pile wall section built on a metro station in Melbourne, Australia, during which five thermal tests (tests 1 to 5) were conducted. Tests 1 to 4 each had an activation period of five days, while on test 5 it lasted 28 days, with recovery being monitored, and its duration adjusted accordingly to construction activities. Tests 1, 2, and 5 involved the activation of three piles (P49–51) with a thermal load of 5.5 kW, whereas tests 3 and 4 only pile P51 with a thermal load of 3 kW. The overall experimental setup is schematically depicted in Fig. 1. TRT's datalogger recorded measurements of the carrier fluid temperature, flowrate and electrical heating power are shown in Fig. 2. Daily maximum and minimum temperature records from the Bureau of Meteorology Olympic Park station (ID: 086338; latitude:−37.83°, longitude: 144.98°, elevation: 7.53 m) [15], located approximately 2.5 km from the site are compared to the inside temperature records of the station in Fig. 3. Soil's temperature measurements along two subhorizontal boreholes (B.H.) at 8 and 16 m depths are also provided. Figs. 4 and 5 present the temperature and uniaxial strains in the instrumented piles No 50 and 51 (i.e. P50 and P51) at different depths and locations (i.e. towards the excavation-E and ground-G). All times and dates reported refer to Melbourne, Australia local time (UTC+10). All raw data is provided on the supplementary material available on the repository link.

## 4 Experimental Design, Materials and Methods

Three contiguous and equally spaced concrete energy piles were built in the Melbourne geological unit and are used as a case study. The piles incorporate individual pipe circuits connected in parallel to a manifold, which is operated at the surface, and a set of temperature and temperature-strain meters as schematically shown in Fig. 1. Each pile was equipped with four U-shape pipe loops made of High-Density Polyethylene (HDPE), fixed to the reinforcement cage, and radially distributed around half of the pile diameter towards the ground side. Twenty temperature-strains sensors were installed in two piles named P50 and P51 and distributed along the piles as opposing pairs on the ground and excavation side of the pile. Before thermal testing, the pipes were purged, flushed, and pressure tested to ensure the integrity of the pipe circuits. Two 2U-loops in P51 reported pressure drops during testing, leading to two operational loops within the pile. Details on the site conditions, material properties, pipe layout, sensor distribution, relevant dates, and construction stages are described in [1,5,12].

The TRT unit was used to emulate thermal loading on the piles, wherein resultant responses were monitored and periodically recorded at different locations. The unit incorporates four electrical power heaters, a circulation pump, and built-in sensors. The carrier fluid (i.e. water) was circulated along the pipes within the section (P49–51) over two days before manually activating the electrical heaters to register the initial average temperature of the piles. Throughout the tests, the fluid temperature, flowrate, and electrical power (refer to Fig. 2) were recorded at the TRT's datalogger at a 10-minute frequency. Temperature sensors within the station, the soil at the subhorizontal boreholes, and the piles at the active sister bars were registered at the multichannel datalogger every 30 min (refer to Fig. 3, Fig. 4). Piles' uniaxial strains at the sister bars were logged using the same device and sampling frequency (refer to Fig. 5). Further details on the piles' activation are provided in [1].

## Limitations

The dataset is limited to the number of operational sensors as reported herein, the duration of testing, and the fact that measurements were conducted during the construction of the station should be considered when interpreting the data.

## Ethics Statement

The authors have read and followed the ethical requirements for publication. This work does not involve human subjects, animal experiments, or data collected from social media platforms. Therefore, Ethics approvals are not required.

## Credit Author Statement

**Luis Villegas:** Conceptualisation; Investigation; Data curation; Visualisation; Methodology; Writing – Original draft. **Raul Fuentes:** Supervision; Writing – review and editing. **Guillermo A. Narsilio:** Supervision; Data curation; Funding acquisition; Writing - review and editing.

## Data Availability

FigshareExperimental dataset on the thermo-mechanical response of an energy piled wall section (Original data)
